# Long-Term Outcomes of Calcaneocuboid Preservation Following Medial Double Arthrodesis for Rigid Flatfoot: A Minimum 10-Year Evaluation

**DOI:** 10.3390/jcm15113991

**Published:** 2026-05-22

**Authors:** Samuel Laurent, Rayane Benhenneda, Ramy Samargandi, Jean Brilhault

**Affiliations:** 1 Department of Orthopedic Surgery, Centre Hospitalier Régional Universitaire (CHRU) de Tours, 37170 Tours, France; 2 Department of Orthopedic Surgery, Centre Hospitalier de Blois, 41000 Blois, France; 3 Department of Orthopedic Surgery, College of Medicine, University of Jeddah, Jeddah 23218, Saudi Arabia; 4 Centre de la Cheville et du Pied, Clinique Saint Léonard, 49800 Trélazé, France

**Keywords:** double arthrodesis, hindfoot arthrodesis, rigid flatfoot, calcaneocuboidal arthrodiastasis, talonavicular joint, subtalar joint

## Abstract

**Background**: Hindfoot arthrodesis is commonly used to treat rigid flatfoot. While traditionally performed as a triple arthrodesis, recent evidence supports medial double arthrodesis, involving only the talonavicular and talocalcaneal joints and sparing the calcaneocuboid (CC) joint. This study aims to evaluate the long-term condition of the spared CC joint more than 10 years after double arthrodesis. **Methods**: We retrospectively reviewed 17 feet in 13 patients (8 women, 5 men; mean age 43.8 ± 16.5 years) who underwent double arthrodesis for rigid flatfoot. Clinical outcomes were assessed using the AOFAS score and a visual analog pain scale. Radiographic evaluation of the CC joint was based on Graves’ four-stage classification, preoperatively and at final follow-up (minimum 10 years). **Results:** The mean follow-up was 154.6 ± 20.4 months. No patient required additional CC arthrodesis, and all CC joints remained asymptomatic. Radiographically, degeneration improved in 9 cases, was stable in 6, and worsened in 2. The mean AOFAS score significantly improved from 23.7 ± 9.3 preoperatively to 70.1 ± 9.3 at the longest follow-up (*p* < 0.0001). Similarly, the mean VAS pain score decreased from 6.4 ± 1.3 preoperatively to 2.1 ± 1.2 at follow-up (*p* < 0.0001). **Conclusions:** Double medial arthrodesis spares the CC joint, enabling arthrodiastasis and subchondral remodeling over time. This contributes to long-term pain relief and improved radiographic outcomes. Preserving minimal motion at the CC joint may enhance foot adaptability to uneven terrain and reduce the need for further surgical intervention.

## 1. Introduction

Rigid flatfoot deformity in adults represents a challenging condition that often necessitates surgical intervention, particularly when conservative measures fail to relieve pain or restore function. Flatfoot deformity has been recognized for centuries and remains one of the most extensively studied conditions in foot and ankle surgery. Historical descriptions of pes planus date back to ancient civilizations, while progressive advances in anatomy, biomechanics, imaging, and reconstructive surgery have continuously refined the understanding and management of this complex deformity, leading to the evolution of treatment strategies from conservative and orthotic approaches to modern reconstructive and fusion procedures [1]. Arthrodesis remains a widely accepted procedure for correcting severe deformities and alleviating symptoms by stabilizing the hindfoot and restoring proper alignment [2].

Triple arthrodesis, involving the subtalar, talonavicular, and calcaneocuboid (CC) joints, has traditionally been considered the standard surgical treatment for rigid flatfoot [3,4]. However, concern has grown over time regarding the potential for degenerative changes in adjacent joints following fusion procedures [5,6]. Although triple arthrodesis achieves reliable deformity correction, the inclusion of the lateral column (CC joint) may further reduce the already limited residual mobility of the hindfoot. Previous studies have shown that CC arthrodesis significantly alters hindfoot motion and affects the mobility of adjacent joints, thereby limiting the residual adaptability of the foot during gait, particularly during the mid-stance and propulsion phases, and increasing stress transfer to adjacent articulations, which may accelerate adjacent joint degeneration over time [7,8,9,10,11,12].

As a result, selective fusion procedures such as double medial arthrodesis, which includes only the talonavicular and subtalar joints, have gained interest. By sparing the CC joint, this approach preserves some degree of lateral column mobility and offers biomechanical advantages, such as improved shock absorption and adaptation to uneven surfaces [8,13,14]. Moreover, studies have demonstrated that double medial arthrodesis achieves deformity correction comparable to triple arthrodesis while reducing operative morbidity and preserving foot flexibility [14,15,16].

In particular, distraction of the CC joint, referred to as arthrodiastasis, has been observed as a consequence of medial realignment. This phenomenon may reduce joint loading and promote remodeling of the subchondral bone, thereby potentially limiting degenerative changes over time [17]. Several studies have reported promising short-term outcomes of medial arthrodesis with CC joint preservation [13,14,15,16,18]. However, data on long-term outcomes remain limited.

The aim of this study was to assess the long-term sustainability of sparing the CC joint in rigid flatfoot treated with double medial arthrodesis. Although medial double arthrodesis has shown satisfactory short- and mid-term clinical outcomes, the long-term evolution of the preserved CC joint remains insufficiently documented. In particular, the durability of the arthrodiastasis effect and its potential influence on clinical symptoms and radiographic progression of CC osteoarthritis over time remain unclear. Therefore, we retrospectively analyzed the clinical and radiographic evolution of the preserved CC joint more than ten years after surgery to evaluate the long-term outcomes of this joint-preserving strategy.

## 2. Materials and Methods

This retrospective monocentric cohort study was conducted at a single tertiary center after obtaining ethical approval from the institutional review board for human research (Project No. 2019012). The data were processed in accordance with the MR-004 reference methodology authorization. The main objective of the study was to evaluate, by a retrospective analysis, the evolution of the preserved CC joint in the double medial arthrodesis (talonavicular and subtalar arthrodesis) performed for a rigid flatfoot more than ten years ago. The secondary objectives were to assess radiographic and clinical outcomes. The patients were evaluated regularly during the first year postoperatively (at 3, 6 and 12 months) and then in a personalized manner thereafter. The last radio-clinical analysis was carried out with a minimum follow-up of 10 years.

### 2.1. Inclusion and Exclusion Criteria

Inclusion criteria consisted of adult patients presenting with symptomatic rigid flatfoot deformity who underwent medial double arthrodesis (talonavicular and subtalar joint) with preservation of the CC joint using a standardized surgical technique, with a minimum radio-clinical follow-up of 10 years. Exclusion criteria included concomitant CC arthrodesis due to symptomatic degeneration or advanced osteoarthritis, revision procedures, cases performed using different surgical techniques, and incomplete clinical or radiographic records. Patients unable to participate in long-term evaluation because of severe cognitive impairment, bedridden status, or follow-up shorter than 10 years were also excluded.

### 2.2. Surgical Technique

**Figure 1:**
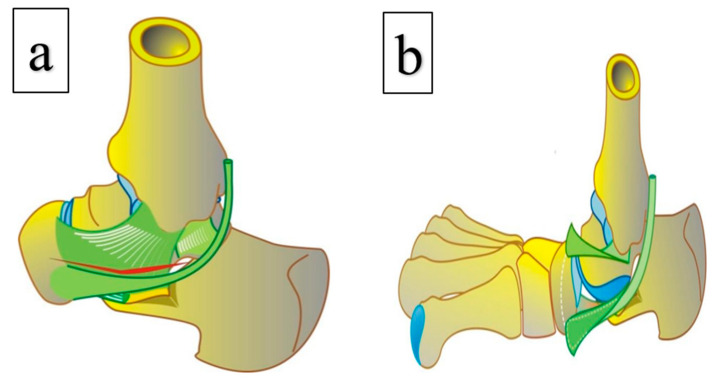
Medial approach centered over the course of the tibialis posterior tendon (**a**). Detachment of the tibialis posterior tendon from the medial aspect of the navicular bone (**b**).

The operative technique was published in 2009 [13]. All patients were operated on by a single senior surgeon. This technique represented the standard institutional practice throughout the study period, allowing treatment of all included patients using a standardized surgical approach. The intervention was performed under regional blocks associated with general anesthesia. The medial ankle approach adjacent to the tibialis posterior pathway was carried out. It extended from the medial malleolus to the medial cuneiform (Figure 1a). This approach exposes the talonavicular joint directly without the need for a large dissection. The arthrotomy was longitudinal, allowing subperiosteal exposure of the talonavicular and subtalar joints. The remain of the tibialis posterior tendon insertion was detached from the medial border of the navicular and moved toward the plantar side (Figure 1b).

**Figure 2:**
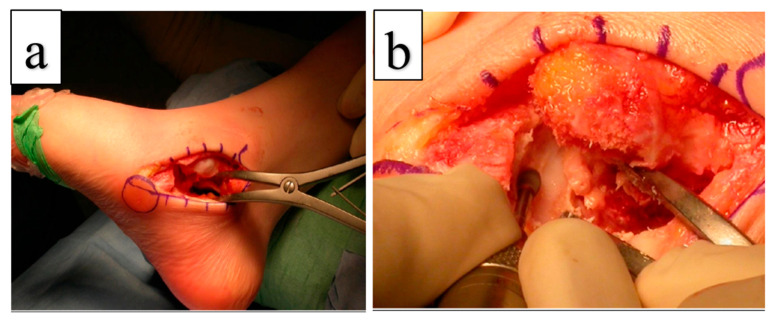
Exposure and preparation of the subtalar joint (**a**). Preparation of the talonavicular articular surfaces down to the subchondral bone (**b**).

The anterior and middle subtalar joints were identified and then, the section of the interosseous ligament was performed to allow the exposure of the posterior subtalar joint. A distractor was positioned under the talar neck to optimize exposure and the preparation of the subtalar joint was carried out (Figure 2a). Once joint preparation was done, a pin distractor was placed between the talus neck and the navicular to expose and prepare the talonavicular joint. The articular surfaces were freshened down to the subchondral bone, respecting their contours by high-speed burr and perforation (Figure 2b).

**Figure 3:**
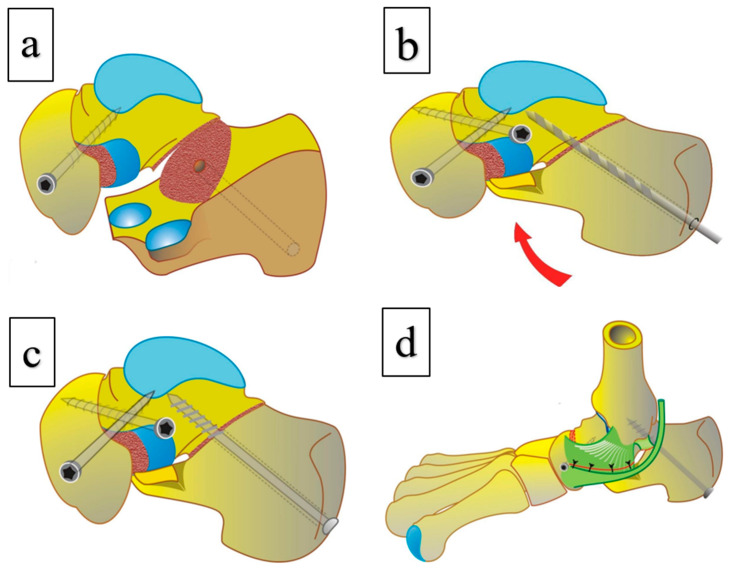
Retrograde compression screw fixation of the talonavicular joint using a 6.5 mm cancellous screw (**a**). Second screw placed for neutralization (**b**). Compression screw fixation of the subtalar joint from the heel into the body of the talus (**c**). Closure in two layers (**d**).

The talonavicular joint was reduced first, which allowed correction of the abduction and pronation of the forefoot. This maneuver brought the calcaneus with it (owing to preservation of the subtalar joint), thereby correcting the abnormal talocalcaneal divergence. The hindfoot valgus was then adjusted. Temporary fixation was done using k-wires. The quality of the correction was assessed by clinical and fluoroscopic analyses (dorsoplantar view, lateral view, and a simulated weight-bearing AP view). The final fixation began with a retrograde screwing for compression of the talonavicular joint (cancellous screw 6.5 mm diameter) (Figure 3a). A second screw (or a staple) was placed for neutralization (Figure 3b). The subtalar arthrodesis was done by a screw from the heel by a compression screw (cancellous screw 6.5 mm in diameter) anchoring in the body of the talus (Figure 3c). In the case of residual bone substance loss in the subtalar space, the bone filling was carried out by iliac cancellous autograft or bone substitute (Triosite^®^, Triosite®, Zimmer Ltd., Swindon, UK). The wound closure was carried out in two planes on a suction drain. The deep plane included the talonavicular articular capsule, the remains of the posterior tibial tendon and especially the periosteum (Figure 3d).

In case an additional procedure in the forefoot is needed, it was performed at the same operating time if it could be performed within the limit of tourniquet time (limited to 120 min); otherwise, it was performed during a second intervention scheduled between 4 and 6 weeks so as not to prolong the period of care. Immobilization by posterior splint was performed after releasing the tourniquet. It was replaced by a plaster cast made on the third postoperative day after checking the wound. The immobilization with non-weight bearing is kept for 6 weeks. At 6 weeks postoperatively, the progressive resumption of weight-bearing was authorized with the abandonment of crutches in the third month postoperatively.

### 2.3. Radiographic Evaluation

**Figure 4:**
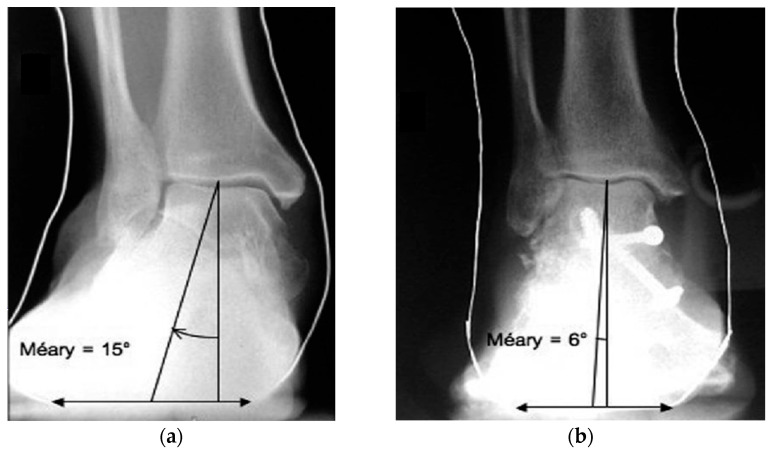
Hindfoot angle (Méary) measured on frontal weight-bearing radiographs with hindfoot alignment view. Preoperative view (**a**) and postoperative view at final follow-up (**b**).

**Figure 5:**
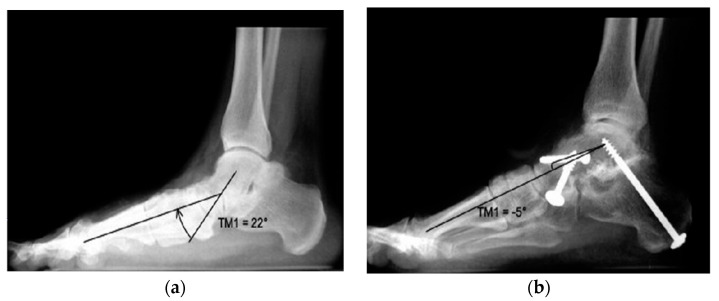
Lateral weight-bearing radiographs demonstrating the talo–first metatarsal (Talo-M1) angle. Preoperative view (**a**) and postoperative view at final follow-up (**b**).

**Figure 6:**
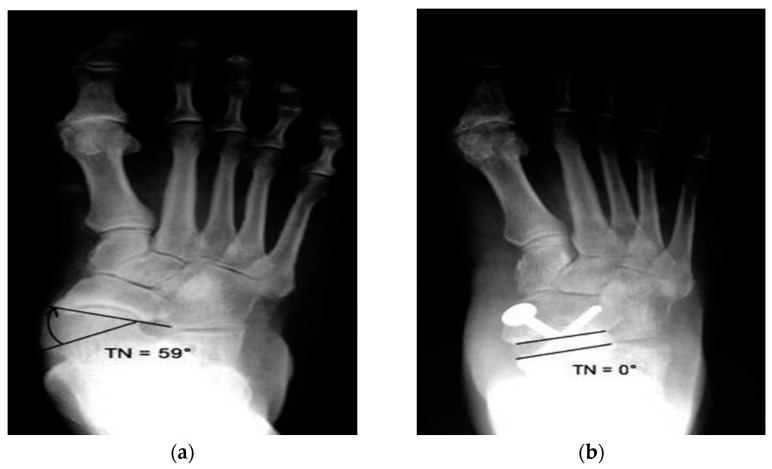
Talonavicular coverage angle measured on anteroposterior weight-bearing radiographs. Preoperative view (**a**) and postoperative view at final follow-up (**b**).

Radiographic assessments included standardized weight-bearing dorsoplantar, lateral, and anteroposterior ankle (Méary view) radiographs. The evaluation of the CC joint degeneration was carried out on the basis of the four-stage score proposed by Graves et al. [19] preoperatively and at the last follow-up: stage 0, arthritic change not appreciable; stage 1, narrowing of the joint space; stage 2, moderate subchondral sclerosis with osteophytes; and stage 3, severe sclerosis with significant narrowing and osteophyte formation. Additional radiologic parameters included the talo–first metatarsal angle (Méary angle) on weight-bearing lateral views, the talonavicular coverage angle or Giannestras angle (on AP dosoplantar views), hindfoot valgus angle (on AP Méary weight-bearing view), and the CC angle (Figure 4, Figure 5 and Figure 6). Measurements were performed with a digital goniometer integrated into the PACS^®^ software (Carestream Health, Rochester, NY, USA; version 11.4.1.0324).

### 2.4. Clinical Evaluation

Clinical outcomes were evaluated using the American Orthopedic Foot and Ankle Society (AOFAS) Score. The objective pain assessment was evaluated using the Visual Analog Scale (VAS). The clinical evaluation looked for spontaneous pain or at the mobilization of the CC joint. Bilateral cases were treated as independent observations.

### 2.5. Statistical Analysis

All data were analyzed using SPSS version 25.0 (IBM Corp., Armonk, NY, USA). Descriptive statistics were reported as means and standard deviations for continuous variables and as percentages for categorical variables. Paired comparisons between preoperative and final follow-up values were conducted using the Wilcoxon signed-rank test. Statistical significance was set at a *p*-value < 0.05. Monte Carlo simulation with 5000 iterations was used to estimate 99% confidence intervals.

## 3. Results

A total of 33 feet in 27 patients were initially identified. Eight patients died during the follow-up period, including one bilateral case and one patient who had a final radio-clinical follow-up exceeding 10 years before death. Four feet were excluded because a concomitant CC joint arthrodesis had been performed due to symptomatic CC joint degeneration and/or advanced osteoarthritis, making these cases unsuitable for joint-preserving surgery. Three additional feet could not be evaluated due to poor general health (bedridden status or significant cognitive impairment). As a result, 17 feet in 13 patients (8 women and 5 men) were included in the final analysis.

The mean age at the time of the intervention was 43.8 years ± 16.53 (range 18–69). The average follow-up was 154.5 ± 20.4 months (range 125–187). The mean body mass index (BMI) was 27.4 ± 7.1 kg/m^2^ (range: 20.3–42.2). The etiologies were for eight neurological cases (cerebral palsy, sequelae of stroke, paraplegia due to post-traumatic spine injury), for six degenerative cases (posterior hamstring insufficiency, rheumatoid arthritis) and for three post-traumatic cases (two of these traumas occurred in the context of a work accident). A percutaneous lengthening of the Achilles tendon was performed in 14 cases. A peroneus brevis tenotomy was necessary in six cases (Table 1).

The arthrodesis was carried out by two screws with a diameter of 6.5 (one in compression, the other in neutralization) for talonavicular fixation (cross screwing) and one screw of diameter 6.5 mm with long thread for subtalar arthrodesis. The occurrence of a bony fracture fragmentation of the talus during cross-screwing required the use of fixation by a plate with integral screws in DARCO titanium (2 screws in the navicular and 2 screws in the neck of the talus). In five cases, the filling of the residual bone gap required the use of a bone substitute (Triosite®, Zimmer Ltd., Swindon, UK). Iliac crest autograft was necessary in two cases. The follow-up was uncomplicated for 15 of the 17 operated patients. One of the cases (post-traumatic flatfoot in a smoking patient) presented with a heel pressure ulcer that required surgical re-operation for skin coverage with a pedicled flap. Another case (post-traumatic flatfoot in a chronic heavy smoker patient) presented with a postoperative infection that required surgical re-intervention for lavage and removal screws relayed by the placement of an external fixator. These two cases had in common chronic tobacco consumption, as well as a precarious skin condition prior to surgery due to a history of defenestration trauma for one and crush injury for the other patient. No other complications were observed. The bone union of these arthrodeses was obtained in all cases during the six months following surgery. All the cases presented an improvement in the alignment of the hindfoot, their pain intensity, their walking perimeter and during shoe wear.

At the longest follow-up, all the cases reviewed had an asymptomatic CC joint. The radiographic evaluation of CC joint degeneration according to the score of Graves et al. [19] showed degeneration improvement in nine feet (1 improved by two stages, 8 by one stage), six cases had not progressed, and two cases deteriorated by one stage (Table 2).

**Figure 7:**
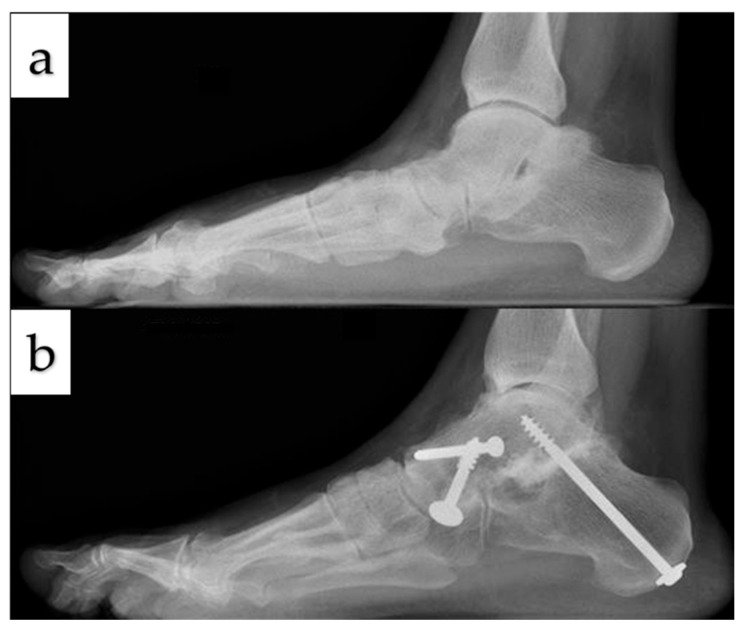
Preoperative radiographs (**a**) and radiographs obtained at final follow-up >10 years (**b**).

**Figure 8:**
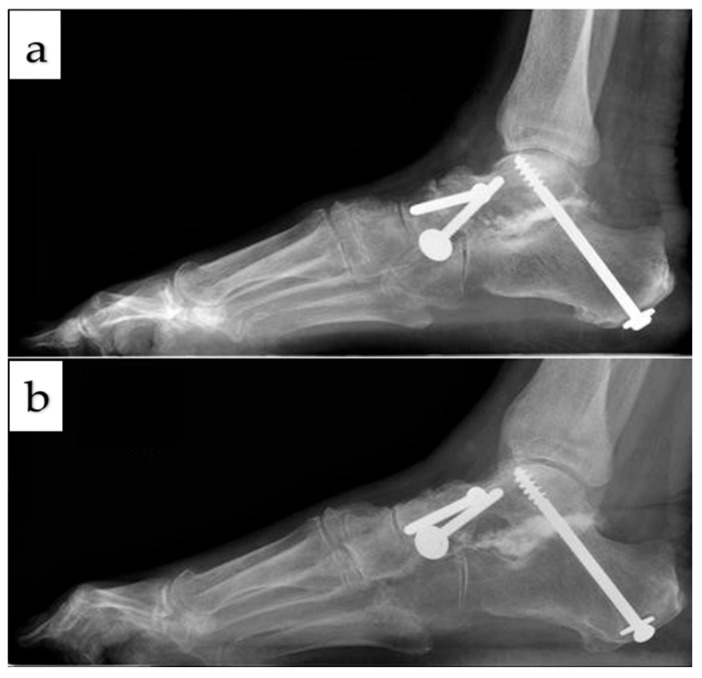
Follow-up radiographs at >12 months (**a**) and at final follow-up >10 years (**b**).

At the longest follow-up, 14 cases presented with an osteoarthritis score of ≤1, while only three had a score of ≥2. (Table 3). Overall, 15 of the 17 cases demonstrated either stabilization or radiographic improvement of CC joint osteoarthritis (Figure 7 and Figure 8).

### 3.1. Clinical Results

The mean AOFAS score improved from 23.7 ± 9.3 (range: 9–43) preoperatively to 70.1 ± 9.2 (range: 52–87) at the longest follow-up (*p* < 0.0001) (Table 4). After surgery, eight patients (10 cases) could use commercial shoes. Patients with rigid flatfoot due to a neurological etiology maintained the use of orthopedic shoes but with better ankle stability.

### 3.2. Radiographic Results

Significant radiographic improvements were observed for the various measurements carried out (Table 5). The mean talonavicular coverage angle increased from 36.5° (24–49°) preoperatively to 17.6° (0–28°) at the longest follow-up (*p* < 0.0001). The mean Talo-M1 angle decreased from 15.2° (4–35°) preoperatively to 1.8° (0–10°) at the longest follow-up (*p* < 0.0001). The mean hindfoot angle (Méary) decreased from 12.9° (4–24°) preoperatively to 7.4° (0–16°) at the longest follow-up (*p* < 0.012). The mean CC angle decreased from 148° (130–168°) preoperatively to 163.8° (150–180°) at the longest follow-up (*p* < 0.0012).

## 4. Discussion

The joint sparing of the lateral column of the foot enabled by the double medial arthrodesis of the hindfoot by a single medial approach is accompanied by a CC arthrodiastasis secondary to the realignment of the talonavicular joint. This distraction allows, in the long term, a remodeling of the subchondral bone, rendering clinically by a regression of the symptoms of pain, and radiologically by an improvement of the stage of osteoarthritis of this joint. Although only a few degrees of movement remain at the CC joint, this reduces the incidence of pain and provides better adaptation of the foot to uneven terrain.

In a study published on 20 cases operated on by a single double medial arthrodesis with an average follow-up of 9.2 months (6–21 months), Berlet et al. [14] had identified the frequency of decompression of the CC joint. The evaluation of the radiographic changes highlighted the existence of a distraction of the CC joint, responsible for an increase in the joint space and an improvement of at least one stage of osteoarthritis in 50% of cases. The evolution was less predictable in severe CC osteoarthritis, with only 20% improvement on radiography [20]. Our experience supports these results. In our series with a greater follow-up of 154.59 ± 20.4 months (range: 125–187), one case presented an improvement of two stages, eight cases an improvement of one stage, six cases remained stable, and two cases worsened by one stage, resulting in an improvement of at least one stage of CC arthritis in 53% of operated cases. At the last follow-up, no foot was reoperated for a complementary CC arthrodesis, and all the CC joints were asymptomatic. These results are comparable to other series in the literature [13,14,15,21]. Biomechanical studies on cadavers have shown that most movements at the CC joint are suppressed by the talonavicular fixation. Only a few degrees of movement remain [8]. These remaining degrees of motion help dissipate the wandering forces exerted on adjacent joints. The abduction correction through the TN joint provides an arthrodiastasis effect on the CC joint, reducing joint pressure and allowing remodeling of the subchondral bone [14]. In our study, the radiological measurements confirm the presence of an arthrodiastasis and its maintenance over time. These phenomena of joint distraction and subchondral remodeling are well described in the ankle [17].

In his series of 16 cases (14 patients), Sammarco [8] reported the radio-clinical results of double arthrodesis by a medial and lateral approach for symptomatic flatfoot, with a minimum follow-up of 18 months (18–93 months). The correction obtained was comparable to that reported in primary triple arthrodesis performed by a double approach. Subtalar and talonavicular fusion were obtained in all patients except one who had a talonavicular non-union at 6 months. The degeneration of the CC joint was evaluated clinically and radiographically using the Graves four-point score. At the longest follow-up, five cases presented radiographic evidence of increased arthritic changes, although none were symptomatic. All cases of increased osteoarthritis of the CC joint occurred in patients with flatfoot, though three of them also had rheumatoid arthritis. In these three cases, the degenerative changes may have been induced as much by rheumatoid arthritis as by the lateral overload of valgus deformity. The authors highlighted that arthritis symptoms could appear 15 years or more after arthrodesis. Our results support the protective effect of the residual movement in the CC joint on adjacent articulations.

Our series showed a significant improvement in the average AOFAS score, which increased from 23.7 ± 9.3 (range: 9–43) preoperatively to 70.1 ± 9.2 (range: 52–87) at the longest follow-up (*p* < 0.0001), demonstrating the effectiveness of double medial arthrodesis on pain relief and quality of life, regardless of preoperative foot morphology. These results are comparable to other series in the literature [8,13], especially since the same clinical evaluation criteria were applied. An improvement in shoe-wearing ability was reported in 10 cases, allowing the use of commercial footwear. Patients with neurological flatfoot retained orthopedic shoes but experienced improved ankle stability. This clinical improvement was accompanied by significant improvement in various radiological parameters.

Radiographic analysis showed significant correction of the hindfoot alignment, regardless of the initial preoperative deformity. The correction observed in our study was equivalent to results from the previous literature evaluating double medial arthrodesis via a single medial approach [13,18,22] and also comparable to those achieved with triple arthrodesis [3,23,24,25,26]. DeVries et al. [16] found no significant differences in deformity correction between double and triple arthrodesis, both yielding radiographic results close to normal. Similarly, Hyer et al. [18] observed a 3.64 times higher likelihood of persistent ankle valgus in patients who underwent triple arthrodesis compared to those treated with double medial arthrodesis.

The literature increasingly supports double arthrodesis as a reliable technique for rigid flatfoot correction. In our series, double medial arthrodesis resulted in significant clinical and radiographic improvement with a low rate of major complications. These findings are consistent with the literature reporting fusion rates and time to union comparable to triple arthrodesis, while offering additional advantages such as reduced operative time, fewer soft tissue complications, and lower risk of peri-articular degeneration [20,25,27,28,29]. Cates et al. [27] reported nearly identical fusion rates and time to union between double and triple arthrodesis, whereas Galli et al. [30] demonstrated that medial double arthrodesis is more cost-effective and efficient, with significantly reduced operative duration and hardware expenses. This technique has additional surgical advantages, including adequate joint preparation [31], excellent deformity correction [22,28], reduced operative time [29,30], satisfactory fusion rates [7,28,29], and fewer soft tissue complications [7,13,31].

Importantly, the two major complications observed in our series occurred in chronic heavy smokers with compromised soft tissue envelopes secondary to previous trauma. These findings emphasize the importance of careful patient selection and preoperative soft tissue assessment before undertaking hindfoot arthrodesis, particularly in patients with vascular compromise, smoking history, or poor skin quality. In such cases, smoking cessation and meticulous perioperative management should be strongly encouraged to reduce the risk of postoperative complications.

In parallel, contemporary foot and ankle surgery increasingly favors joint-preserving and soft tissue-preserving procedures whenever feasible. A recent systematic review by Vaggi et al. [32] highlighted the growing role of minimally invasive approaches, such as medial displacement calcaneal osteotomy, in flexible Stage II deformities. However, rigid Stage III deformities still generally require corrective arthrodesis to achieve durable alignment correction and pain relief. In this context, medial double arthrodesis may represent a valuable compromise between reliable deformity correction, preservation of residual lateral column function, and limitation of soft tissue morbidity compared with more extensive fusion procedures.

This study has certain limitations. It is retrospective and conducted at a single center, with a small sample size and no control group, which may limit the generalizability of the findings. The absence of systematic computed tomography (CT) verification of union, potential recall bias in patient-reported outcomes, and heterogeneity in etiologies also present constraints. Nonetheless, to our knowledge, this is the longest follow-up study to assess the evolution of the CC joint in patients who underwent double medial arthrodesis, providing valuable insights into its long-term effectiveness and joint-preserving potential. Future prospective multicentric studies with larger cohorts and systematic CT evaluation would be valuable to more rigorously assess fusion quality, subchondral remodeling, and long-term evolution of the preserved calcaneocuboid joint.

## 5. Conclusions

The sparing of the CC joint during medial double arthrodesis in the case of a rigid flatfoot causes CC arthrodiastasis, which allows, in the long term, a reshaping of the subchondral plate resulting clinically in a regression of the pain, and radiologically by improving the degree of osteoarthritis of this joint. Although only a few degrees of movement remain at the CC joint, this reduces the incidence of pain and provides better adaptation of the foot to uneven terrain. Particular caution should nevertheless be exercised in heavy smokers and patients with compromised soft tissue conditions, as these factors may increase the risk of postoperative wound complications and infection.

## Figures and Tables

**Table 1:** Demographic and Surgical Characteristics of the Study Population.

Variable	Value
**Number of cases (number of patients)**	17 cases (13 patients)
**Sex**	
– Male	5 (38.5%)
– Female	8 (61.5%)
**Age (years)**	43.8 ± 16.5 [18–69]
**BMI (kg/m^2^)**	27.4 ± 7.18 [20.3–42.2]
**Site**	
– Left	7 (41.1%)
– Right	10 (58.8%)
**Etiology**	
– Neurological	8 (47.1%)
– Degenerative	6 (35.3%)
– Post-traumatic	3 (17.6%)
**Associated Surgery**	
– Achilles tendon lengthening	14 (82.4%)
– Peroneus brevis tenotomy	6 (35.3%)
**Follow-up (months)**	154.6 ± 20.4 [125–187]

**Table 2:** Evolution of the stage of osteoarthritis at the last follow-up.

Variation in Unit of the Score of Graves et al. [19]	Number of Cases	Percentage (%)	Cumulated (%)
−2	1	6%	6%
−1	8	47%	53%
0	6	35%	88%
1	2	12%	100%
Total	17	100%	

**Table 3:** Distribution of cases at the last follow-up, according to the score of Graves et al. [19].

Score of Graves et al. [19]	Number of Cases at >10 Years	Percentage (%)	Cumulated (%)
0	8	47%	47%
1	6	35%	82%
2	2	12%	94%
3	1	6%	100%
Total	17	100%	

**Table 4:** Preoperative and Longest Follow-up (>10 years) AOFAS Scores.

AOFAS																				
Case N°	Pain		Activity		Walk Distance		Walk Surface		Gait		Sagittal Motion		Hindfoot Motion		Stability		Alignment		Score	
	PO	FU	PO	FU	PO	FU	PO	FU	PO	FU	PO	FU	PO	FU	PO	FU	PO	FU	PO	FU
1	20	30	0	4	0	4	0	3	0	4	4	4	3	3	0	8	0	10	27	70
2	20	30	0	7	0	5	0	3	0	4	0	4	0	3	8	8	5	5	33	69
3	0	20	0	4	0	0	0	3	0	4	8	8	6	6	8	8	5	10	27	63
4	0	30	0	7	0	4	0	3	0	4	4	8	3	3	8	8	0	10	15	77
5	20	40	4	10	2	5	0	3	0	4	4	4	0	3	0	8	0	10	30	87
6	20	40	4	10	2	5	0	3	0	4	4	4	0	3	0	8	0	10	30	87
7	20	30	4	7	0	4	0	3	4	8	4	4	3	3	8	8	0	10	43	77
8	0	30	4	7	2	5	0	3	0	4	0	4	3	3	0	8	0	10	9	74
9	0	30	0	4	0	4	3	3	0	4	0	4	0	3	8	8	0	5	11	65
10	0	30	0	4	0	4	3	3	0	4	0	4	0	3	8	8	0	5	11	65
11	0	20	0	4	0	2	0	3	0	0	4	4	3	6	8	8	5	5	20	52
12	0	20	4	7	0	2	3	3	0	4	4	8	3	3	8	8	5	5	27	60
13	0	20	4	7	0	2	3	3	0	4	4	8	3	3	8	8	5	5	27	60
14	20	40	0	4	0	2	0	3	0	4	4	4	3	3	8	8	0	5	35	73
15	0	30	0	4	0	2	0	3	0	4	4	4	3	3	8	8	5	10	20	68
16	0	30	0	4	0	2	0	3	0	4	4	4	3	3	8	8	5	10	20	68
17	0	30	0	7	0	4	3	3	0	4	4	8	3	3	8	8	0	10	18	77

AOFAS: American Orthopedic Foot and Ankle Society score; PO: preoperative; FU: follow-up (>10 years).

**Table 5:** Comparative clinical and radiographic results.

Results	Clinical		Radiographic							
	AOFAS		TN Coverage Angle		Angle Talo-M1		Hindfoot Angle (Méary)		Angle CC	
	PO	FU	PO	FU	PO	FU	PO	FU	PO	FU
Minimum	9	52	24°	0°	4°	0°	4°	0°	130°	150°
Maximum	43	87	49°	28°	35°	10°	24°	16°	168°	180°
Mean	23.7	70.1	36.5°	17.6°	15.2°	1.8°	12.9°	7.4°	148°	163.8°
Standard deviation	9.3	9.3	7.7°	7.3°	10.6°	3°	6.2°	4.9°	11.8°	8.8°
*p* value	<0.0001		<0.0001		<0.0001		<0.012		<0.0012	

AOFAS: American Orthopedic Foot and Ankle Society score; PO: preoperative; FU: follow-up (>10 years); TN coverage angle: talonavicular coverage angle; talo-M1 angle: talo–first metatarsal angle (Méary angle); Hindfoot angle: hindfoot valgus angle; CC angle: calcaneocuboid angle.

## Data Availability

The data presented in this study are available upon request from the corresponding author.
